# Intraoperative Injection of Normal Saline Through Lumbar Drainage for Transnasal Endoscopic Repair of Complex CSF Leaks

**DOI:** 10.3389/fsurg.2022.861406

**Published:** 2022-03-21

**Authors:** Xiaoming Wei, Fan Zhang, Yankai Qiu, Hong Shen, Tatiana Ilyasova, Li Liu

**Affiliations:** ^1^Department of Neurosurgery, The First Affiliated Hospital of Harbin Medical University, Harbin, China; ^2^Department of Neurosurgery, First Affiliated Hospital of Yangtze University, Jingzhou, China; ^3^Department of Anesthesiology, The First Affiliated Hospital of Harbin Medical University, Harbin, China; ^4^Department of Internal Diseases, Bashkir State Medical University, Ufa, Russia

**Keywords:** cerebrospinal fluid leak, normal saline, complex leaks, lumbar drainage, neuroendoscopy

## Abstract

**Objective:**

It is well known that accurate location of the leak in the operation is crucial for repairing cerebrospinal fluid leakage. The study aims to investigate the application of intraoperative injection of normal saline through lumbar drainage in repairing complex leaks.

**Methods:**

The fistulas of all patients with CSF leak were located by computed tomography cisternography (CTC) or heavy T2 magnetic resonance imaging (MRI) before surgery. Before anesthesia, the patient underwent lumbar drainage implantation, and then 20 ml of normal saline was slowly injected through the lumbar drainage to observe the patient's response. The surgical approach was designed based on the preoperative imaging data. When the operation was near to the suspected fistula, normal saline was injected through lumbar drainage (20 ml each time) to confirm the leak location. After CSF leak repair, saline was injected again to confirm whether the repair was successfully.

**Result:**

Of the 5 patients with complex leaks, 4 cases were repaired by transnasal endoscopy method, and 1 case was repaired by transnasal endoscopy method and epidural method. A total of 7 leaks were found during the operation. During the operation, 40–120 ml of normal saline was injected through lumbar drainage. Cauda equina neuralgia was developed in patients who received 120 ml normal saline, which was relieved by intrathecal injection of dexamethasone. During the follow-up of 3 months, 1 case suffered from brain abscess, which was controlled by vancomycin. There was no recurrence of rhinorrhea.

**Conclusion:**

Intraoperative injection of normal saline through lumbar drainage can not only better expose the complex leak but also check the repair effect of the leak during transnasal endoscopic repair, which is effective and avoids side effects.

## Introduction

Precise exposure of cerebrospinal fluid (CSF) leaks and intraoperative verification of satisfactory repair are essential for transnasal repair of CSF leaks (1, 2). Although there are methods to find leaks before surgery (3), it is difficult to identify complex leaks (such as multiple leaks, leaks with multiple repair failures, and high-flow leaks) during surgery. Even with the methods of increasing intracranial pressure such as breath holding and compression of the jugular vein during surgery (4, 5), leaks are not well exposed, which often enhances the difficulty of surgery or even causes repair failure. Although preoperative lumbar injection of fluorescein is the exact method for the intraoperative tracing of leaks (6), it is an off-label surgical medication with the potential side effects (7, 8). In our practice, we performed intraoperative tracing of the leak by intraoperative injection of normal saline through lumbar drainage, which can further verify whether the repair is successful. From October 2019 to June 2021, five patients with complex leaks were treated at the Department of Neurosurgery, the First Affiliated Hospital of Harbin Medical University. Intraoperative injection of normal saline through lumbar drainage achieved satisfactory outcomes. The summarized experience is as follows.

## Methods

1. The fistulas of all patients with CSF leak were located by computed tomography cisternography (CTC) or heavy T2 magnetic resonance imaging (MRI) before surgery (9).

2. Before anesthesia, the patient underwent lumbar drainage implantation, and then 20 ml of normal saline was slowly injected through the lumbar drainage to observe the patient's response. If the patient had no obvious discomfort, normal saline was injected into the lumbar cistern during the operation; Otherwise, normal saline injection will not be performed.

3. The surgical approach was designed based on the preoperative imaging data. When the operation was near to the suspected fistula, the fistula was searched with endoscope,0 degree or 30 degree, 4 mm in diameter, and 18 cm in length (Karl Storz GmbH & Co KG, Tuttlingen, Germany), and then normal saline was injected through lumbar drainage (20 ml each time) to confirm the leak location. Using different repair methods, the leakage was repaired mainly by “bath-plug method” and nasal septal mucosal flap (10, 11). After successful repair, saline was injected again to confirm whether the repair was successfully.

## Result

From October 2019 to June 2021, 5 patients of complex leaks were suffered from injection of normal saline through lumbar drainage. Demographic and clinical data of the patients are listed in Table 1. Of the 5 patients, 3 had traumatic CSF rhinorrhea and 2 had spontaneous CSF rhinorrhea. Of the 5 patients with complex leaks, 4 cases were repaired by transnasal endoscopy repair, and 1 case was repaired by transnasal endoscopy repair and epidural repair (Table 1). A total of 7 leaks were found during the operation, including 1 in tuberculum sellae of sphenoid sinus, 1 in frontal sinus, 1 in ethmoid plate, 1 in lateral recess of sphenoid sinus and 3 in ethmoid sinus roof. During the operation, 40–120 ml of normal saline was injected through lumbar drainage. Cauda equina neuralgia was developed in patients who received 120 mL normal saline, which was relieved by intrathecal injection of dexamethasone. During the follow-up of 3 months, 1 case presented brain abscess, which was controlled by vancomycin. There was no recurrence of rhinorrhea.

**Table 1:** Patient information.

**Case**	**Age**	**Sex**	**Etiology of leak**	**Location of leak**	**Number of operation**	**Complication**
1	35	M	Traumatic	Right tuberculum sellae	3	Cauda equina neuralgia
2	50	F	Spontaneous	Right ethmoid sinus roof	1	-
3	56	M	Traumatic	Right frontal sinus and both ethmoid sinus roofs	1	Brain abscess
4	60	M	Traumatic	Ethmoid plate	1	-
5	30	F	Spontaneous	Lateral recess of sphenoid sinus	1	-

## case Presentation: Case 1

**Figure 1:**
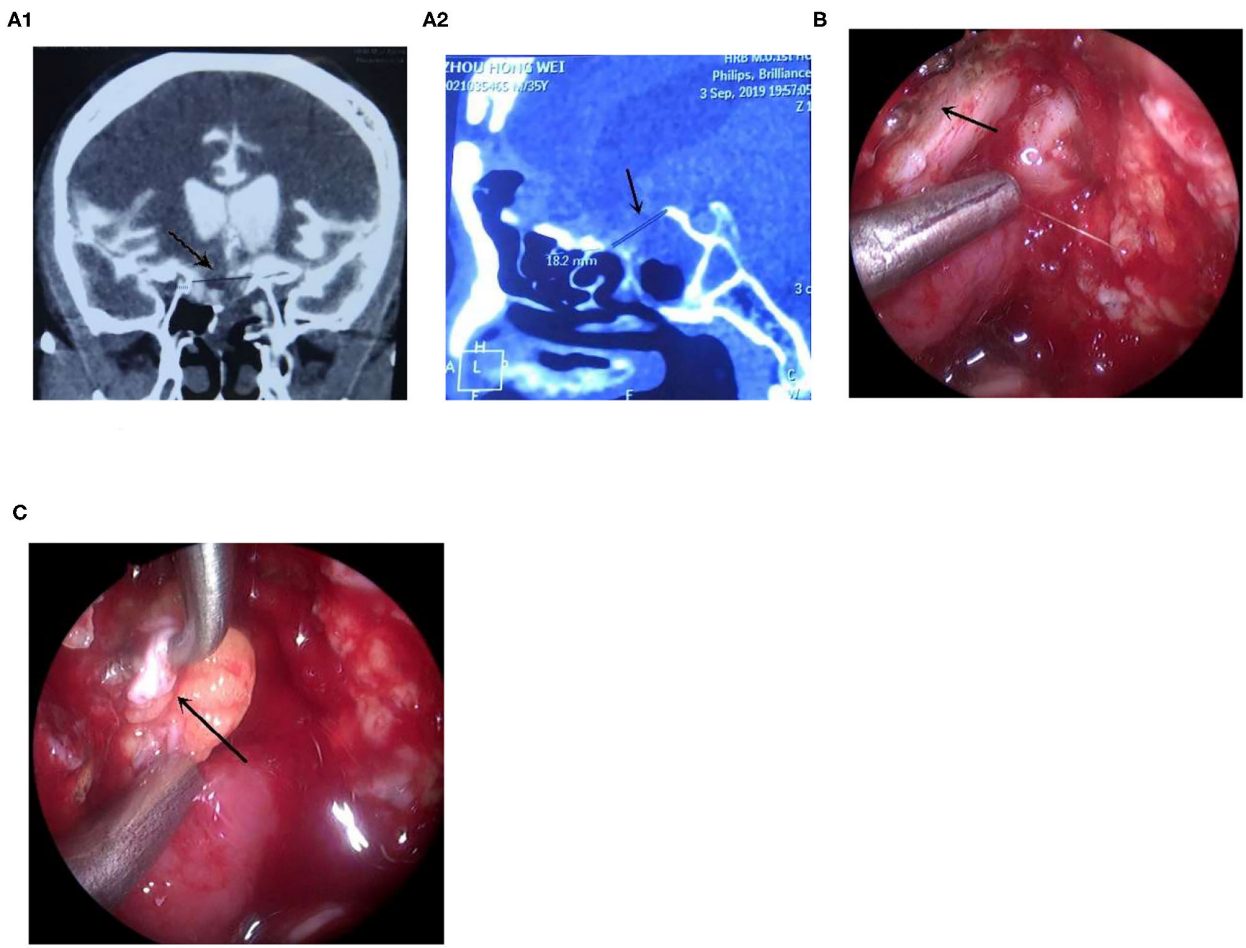
The leak with multiple repair failures was repaired endoscopically. **(A)** The CTC examination showed that the leak was located in the right tuberculum sellae, **(A1)** the coronal CT, **(A2)** the sagittal CT, arrow showed the leak; **(B)** Adequate exposure of the leak after injection of saline; arrow showed the leak. **(C)** The fascia was plugged into the leak using the “bath-plug method,” arrow showed the fascia (Supplementary Video 1).

Case 1 (shown in Figure 1) was a 35-year-old male. The patient had a traffic accident 10 years ago, resulting in skull base fracture and CSF leaks. Five years ago, he had purulent meningitis because of the rhinorrhea, and an anti-inflammatory treatment was carried out followed by craniotomy to repair the leaks. During the surgery, extensive fractures of the anterior fossa floor were observed and repaired with an artificial dura mater. However, intracranial infection occurred due to rhinorrhea both 4 and 2 years ago, and the situations were improved after antibiotic and lumbar drainage treatment. One month ago, he came to our hospital because of rhinorrhea and fever again, and the preoperative CTC examination showed a leak in the right tuberculum sellae of sphenoid sinus. The first transnasal repair showed extensive chronic inflammation of the nasal mucosa and the leak was indeed in the right tuberculum sellae. Due to the large size of the leak, muscle, fascia lata and pedicled nasal septum mucosal flaps were applied to repair the leak after the removal of the sphenoid sinus mucosa. The patient had no recurrence of rhinorrhea. On the 16th day after the surgery, the patient developed fever and rhinorrhea. The second surgery showed infection in the sphenoid sinus and the infected muscle and fascia lata was subsequently removed. The nasal septum mucosal flap was used alone to repair the leak. After the surgery, the patient had no fever or intracranial infection but there was intermittent rhinorrhea. Thus, reoperation was performed 18 days after the second surgery, during which, 100 mL of normal saline was injected through lumbar drainage to further clarify that the CSF leak was located in the right tuberculum sellae. The fascia lata was inserted into the leak using the “bath-plug method” (10). After the successful repair, 20 mL of normal saline was injected into the lumbar cistern to verify whether the repair is satisfactory, and a larger fascia was further applied to the outside of the inserted fascia (Supplementary Video 1). After awakening from anesthesia, the patient developed a cauda equina irritation sign, which was relieved after intrathecal injection of dexamethasone. During the 3-month follow-up, there was no recurrence of rhinorrhea.

## Case Presentation: Case 2

**Figure 2:**
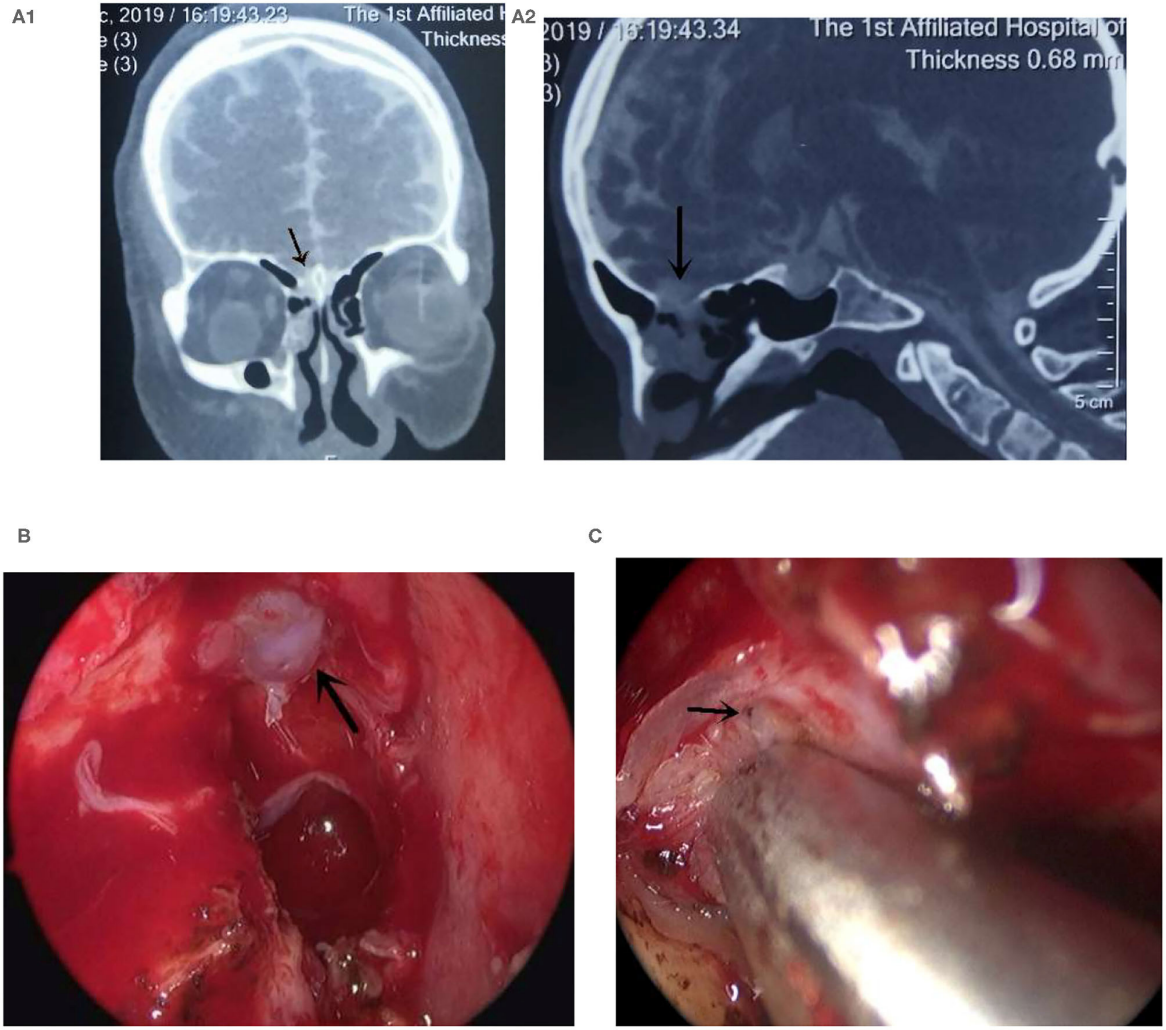
The high-flow leak was repaired endoscopically. **(A)** The CTC examination showed that the leak was located in the right ethmoid pate, **(A1)** the coronal CT, **(A2)** the sagittal CT, arrow showed the leak; **(B)** Arrow showed the edematous mucosa; **(C)** Exposed “pinpoint” leak after the injection of saline through lumbar drainage indicated by the arrow.

Case 2 (shown in Figure 2) was a 50-year-old female with spontaneous rhinorrhea and a previous history of breast cancer. After admission, lumbar drainage was performed, and the patient still had massive rhinorrhea. Thus, it was considered to be a “high-flow” leak. Endoscopic exploration of the leak showed significant mucosal edema, which seemed to “disappear” after the removal of the mucosa. 20 mL of normal saline was injected through lumbar drainage, which showed that the leak was “needle-like” in size. The leak was filled using muscle and fascia followed by injecting 20 mL of normal saline to verify whether the repair is satisfactory before packing and fixation. During the 2-month follow-up, there was no recurrence of rhinorrhea.

## Case Presentation: Case 3

**Figure 3:**
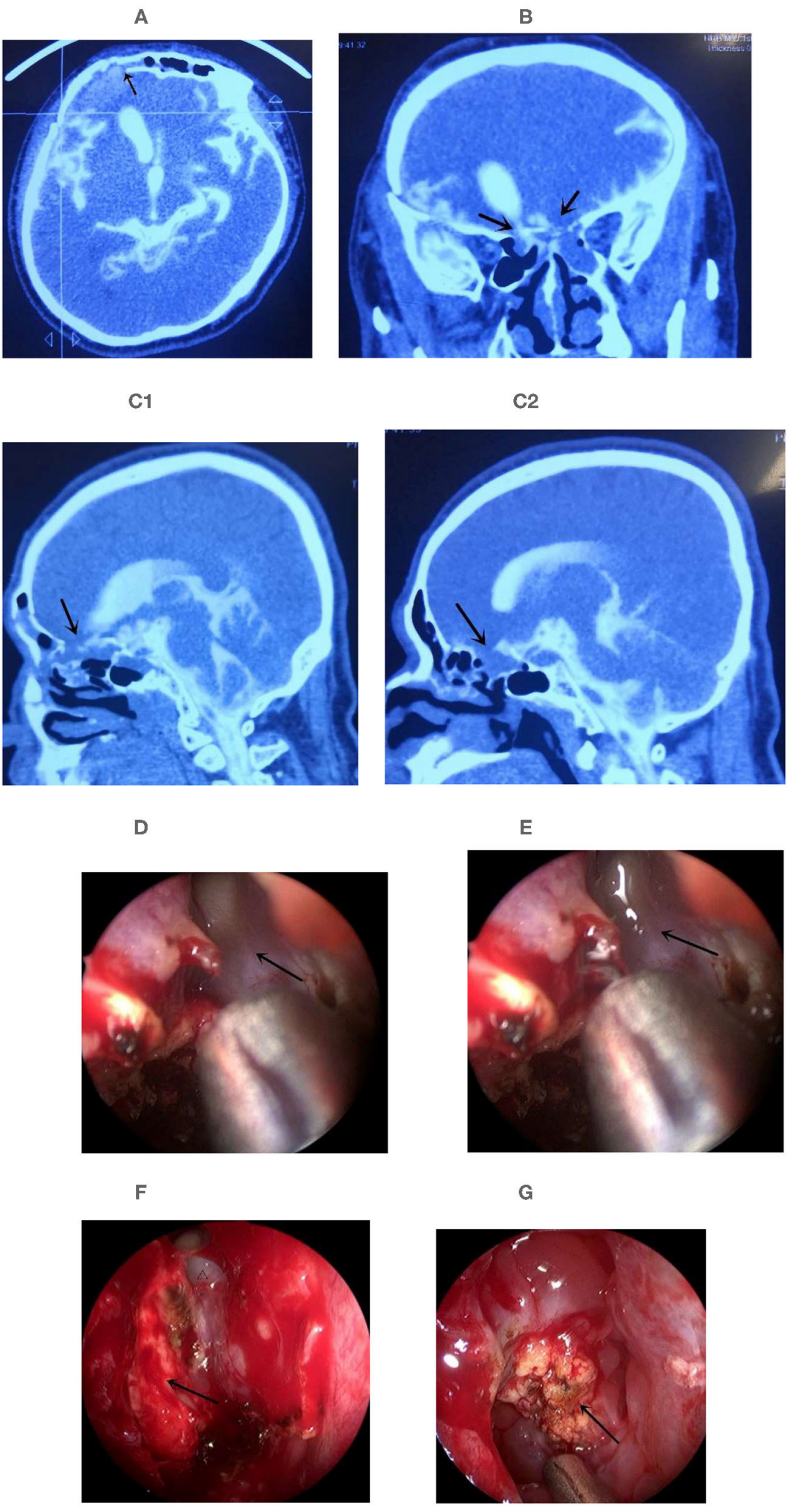
Multiple leaks were repaired by transnasal endoscopy combined with the epidural approach. **(A–C)** The CTC examination located the leaks before surgery. **(A)** The leak was in the right frontal sinus; **(B)** The leak was located in the both ethmoid sinus roofs of the coronal CT. **(C1)** Sagital CT of right ethmoid, **(C2)** Sagital CT of left ethmoid. Arrow show the suspected leak. **(D)** No CSF leak in the right frontal sinus, arrow show the orifice of frontal sinus. **(E)** CSF leak was found in the right frontal sinus when intraoperative injection of 40 mL normal saline through lumbar drainage to expose the leak. Arrow show the CSF in the orifice of frontal sinus. **(F)** Encephaloceles in the right ethmoid sinus roof leakage, arrow show the encephaloceles, triangle indicated the orifice of right frontal sinus; **(G)** Encephaloceles in the left ethmoid sinus roof leakage, arrow show the encephaloceles (Supplementary Video 2).

Case 3 (shown in Figure 3) was a 56-year-old male. He was admitted because of traumatic rhinorrhea. Preoperative CTC showed three suspected leaks: leaks in the right frontal sinus and both ethmoid sinus roofs. Both ethmoid sinus roofs leaks were confirmed during the surgery. The encephaloceles were observed in both ethmoid sinus roofs. The right frontal sinus was demonstrated to be a leak by further injection of saline. When injection saline, the CSF leak was founded, otherwise there was no CSF leak (Supplementary Video 2). The encephaloceles were removed and the leaks were subsequently repaired. The skull defect of the right ethmoid sinus was found to be large, and the two ethmoid sinus roof defect was successfully repaired using thigh adipose tissue and fascia lata followed by injecting 20 mL of normal saline to check the repair. The frontal sinus leak was then repaired by epidural method to close the right frontal sinus by muscle. More than 1 month after discharge, the patient had a brain abscess but no rhinorrhea, which was controlled by vancomycin.

## Discussion

### Shortcomings of Preoperative Leak Location

There are many methods to locate the leak preoperatively, but whether the imaging method is the most efficient remains undetermined. Eljazzar et al. (12) recommended CT combined with MRI to locate the leak after reviewing numerous literature. The preferred method in our center is the CTC combined with three-dimensional fast-imaging employing steady-state acquisition (3D-FIESTA), which has achieved good results in practical applications. However, during the surgery, we found it difficult to discover some leaks due to abnormal anatomy (traumatic lead) and multiple leaks, which we called complex leaks. To further identify the leaks intraoperatively, the previously commonly used methods are intrathecal fluorescein injection, breath holding, and compression of the jugular vein to increase intracranial pressure. There is no doubt about the role of intrathecal fluorescein injection in further locating the leaks during surgery, but it is off-label use (13), and the consequences are unacceptble once complications occur; intraoperative compression of the jugular vein and breath holding can be used to assist in locating the leaks, but the effect is not significant. So, the above methods limit the further identification of complex leaks. Therefore, how to identify these complex leaks faster and more accurately is particularly important.

### Intraoperative Injection of Normal Saline Through Lumbar Drainage

Our experience with the CTC is as follows (9). When the rhinorrhea is not obvious, intrathecal injection of contrast agent followed by injection of 10–20 mL of normal saline can make the patient develop rhinorrhea, achieving the purpose of increasing CTC sensitivity. We assume that the leaks can be exposed intraoperatively by directly increasing the volume of CSF and increasing intracranial pressure with a similar method. It has been reported in the literature that intraoperative injection of normal saline helps fluorescein identify leaks that are more difficult to expose (1, 10). Therefore, we used this method for intraoperative exposure of complex leaks, achieving full exposure and satisfactory repair. However, there is no uniform standard for how much normal saline to inject. It has been reported to be 10–60 mL by Xie et al. (1) and 40–140 mL by Wormald et al. (10). During our surgery of Case 1, 100 mL of saline was injected to fully expose the leak with an abnormal anatomical structure and then 20 mL of saline was injected again to check the repair, with a total of volume of 120 mL. But after awakening from anesthesia, the patient developed cauda equina neuralgia, which was relieved after intrathecal injection of dexamethasone. It is believed that the patient developed this symptom possibly because of excessive saline injection. The treatment experience is as follows: inject 20 mL of normal saline before anesthesia to determine whether there is rhinorrhea and determine the dose of normal saline that needs to be injected at one time during surgery; meanwhile, observe whether there are symptoms of cauda equina stimulation. If yes, the injection of normal saline through lumbar drainage should be slow and the total amount should be small. The symptoms of cauda equina stimulation can be relieved by injecting a small amount of dexamethasone after surgery. In the Case 2, the intraoperative exploration of the skull base, the skull base did not appear to be abnormal, and CSF flowed from the “needle-like” sized leak only after the injection of saline for repair. A total of 40 mL of saline was injected, and there were no postoperative complications. In the other cases, a total of 40–60 mL of saline was injected to expose the leaks adequately without associated complications.

### Compared With Injection of the Fluorescent Agent

We applied the injection of saline through lumbar drainage in the repair of complex CSF leaks. For example, in the case of high-flow leaks or leaks with large defect (more than 1 cm) (14), multiple leaks, and leaks failed to be repaired initially, it is not only possible to find the leaks, but also verify whether the intraoperative repair is satisfactory.

The injection of the fluorescent agent through lumbar drainage is conducted before the start of transnasal endoscopic surgery, so the fluorescent agent can be found when entering the nasal cavity and CSF leaks can be found with the prompt of the fluorescent agent (15, 16). At present, due to preoperative CT cisternography, the accuracy of non-interval magnetic resonance scanning with heavy T2 in judging CSF leaks can reach 95%. Most of the surgeons can determine the initial location of the leak, so it is of little significance to inject the fluorescent contrast agent preoperatively to achieve the detection of the fluorescent contrast agent when entering the nasal cavity.

In our initial detection of the location of the leak and management of structures such as the mucosa around the leak, extensive removal of skull base soft tissue had the risk of further aggravating rhinorrhea due to the patient's extensive skull base defects in Case 1. We only injected normal saline in this case to identify the exact location of the CSF leak, then further injected normal saline after the repair procedure to verify the accuracy of the repair procedure. When multiple leaks were suspected preoperatively, the mucosa around the leak was surgically treated, and the already found leak could not be easily identified due to the patient's reduced cranial pressure. It is very easy for us to identify the leak with certainty by injecting normal saline intraoperatively as in case 3.

We believe that intraoperative injection of normal saline through lumbar drainage is effective and avoids side effects such as spinal cord injury caused by the injection of fluorescent agents through lumbar drainage.

In summary, we believe that the injection of normal saline through lumbar drainage during transnasal endoscopic repair of rhinorrhea, which not only better exposes complex leaks but also verifies the repair effect. However, the sample size in this study is small, which needs to be enlarged to further investigate the advantages and disadvantages.

## Data Availability Statement

The raw data supporting the conclusions of this article will be made available by the authors, without undue reservation.

## Ethics Statement

Ethical review and approval was not required for the study on human participants in accordance with the local legislation and institutional requirements. The patients/participants provided their written informed consent to participate in this study. Written informed consent was obtained from the individual(s) for the publication of any potentially identifiable images or data included in this article.

## Author Contributions

XW, FZ, and LL contributed to conception and design of the study. YQ and HS organized the database. TI wrote section of the manuscript. All authors contributed to manuscript revision, read, and approved the submitted version.

## Funding

This work was supported by Applied Technology Research and Development Program Project of Heilongjiang Provincial Science and Technology Department (GA20C019) and Research Innovation Foundation of First Affiliated Hospital of Harbin Medical University (2020M19).

## Conflict of Interest

The authors declare that the research was conducted in the absence of any commercial or financial relationships that could be construed as a potential conflict of interest.

## Publisher's Note

All claims expressed in this article are solely those of the authors and do not necessarily represent those of their affiliated organizations, or those of the publisher, the editors and the reviewers. Any product that may be evaluated in this article, or claim that may be made by its manufacturer, is not guaranteed or endorsed by the publisher.
